# The relationship between high physical activity and premenstrual syndrome in Japanese female college students

**DOI:** 10.1186/s13102-022-00569-0

**Published:** 2022-09-26

**Authors:** Rika Kawabe, Chang Yu Chen, Saori Morino, Kohei Mukaiyama, Yuki Shinohara, Masaya Kato, Hiroki Shimizu, Kanako Shimoura, Momoko Nagai-Tanima, Tomoki Aoyama

**Affiliations:** 1 Department of Physical Therapy, Human Health Sciences, Graduate School of Medicine, Kyoto University, Kyoto, Japan; 2 Department of Rehabilitation Science, School of Medicine, Osaka Metropolitan University, Osaka, Japan

**Keywords:** Premenstrual syndrome, International Physical Activity Questionnaire, Physical activity, Woman’s health

## Abstract

**Background:**

In recent years, moderate physical activity has attracted the attention of experts and women as a way to cope with premenstrual syndrome (PMS). Studies investigated the effects of exercise on PMS, but only a few reports focused on the relationship between physical activity, which included not only exercise but also routine bodily movements, and PMS. Therefore, the present study investigated the relationship between the amount of physical activity and PMS symptoms among sexually mature female students.

**Methods:**

A total of 381 female university students in Japan were surveyed using a paper or web-based questionnaire with the same content. The questionnaire consisted of basic information, PMS symptoms, and physical activity based on the International Physical Activity Questionnaire (IPAQ). Participants were divided into two groups (≥ 3000 The Metabolic Equivalent of Task (MET)-minutes/week and < 3000 MET-minutes/week) based on their total physical activity as calculated using the IPAQ guidelines. The two groups were then compared in terms of the severity of their PMS physical and psychological symptoms as calculated based on the American College of Obstetricians and Gynecologists’ PMS diagnostic criteria. The Wilcoxon's rank-sum test was used for statistical analyses. We then divided the participants based on the presence or absence of each symptom and used the chi-square test to compare the intergroup differences in ratios. The statistical significance level was set at p < 0.05.

**Results:**

Those with total physical activity of ≥ 3000 MET-minutes/week had lower total PMS symptom scores (*p* < 0.01), physical symptom scores (*p* = 0.01), and psychological symptom scores (*p* = 0.01) compared with those with total physical activity of < 3000 MET-minutes/week.

**Conclusion:**

These results suggest that young women with high physical activity (≥ 3000 MET-minutes/week) have milder symptoms of PMS.

**Supplementary Information:**

The online version contains supplementary material available at 10.1186/s13102-022-00569-0.

## Background

Premenstrual syndrome (PMS) is characterised by emotional, behavioural, and physical symptoms that occur during the late luteal phase of the menstrual cycle [1]. It terminates after the onset of menstruation. Premenstrual dysphoric disorder (PMDD) is a more severe condition that includes functional impairment and disruption of personal relationships, such as depression [2, 3]. There is abundant clinical research on perimenstrual symptoms, which include PMS and menstrual symptoms. The frequency of PMS, including mild symptoms, is estimated to be relatively high (80–90%) [4]. It has been reported that 5–8% of reproductive-aged women exhibited moderate to severe PMS symptoms that interfered with their daily activities [5]. PMS may be caused by a variety of factors, mainly including sex hormones, autonomic nervous system, psychological factors, and lifestyle (sleep, diet, exercise, stress, and personal preferences) [6]. Many women prefer non-medical treatment options for PMS. This is mainly because they want to avoid the side effects, contraindications to drug treatments, and the high cost of treatment [5, 7]. Current recommendations for coping with PMS include exercise [8] and dietary management [9]. These are often cited as a way of managing PMS and are considered important non-pharmacological treatments that can be adjusted accordingly. However, there is a lack of comprehensive research on the relationship between lifestyle and PMS symptoms.

Several studies showed that exercise could improve PMS symptoms. These included aerobic exercises, such as swimming exercises [10]. Dahnavi et al. created an aerobic exercise plan and provided an eight-week intervention, three times a week and 30 min each time for female students, and they suggested that this plan could reduce the physical symptoms of PMS [11]. The intensity was determined using the heart rate, which should be within 120–150 beats per minute (bpm) after exercise. Kamalifard et al. conducted a yoga exercise intervention with women aged 20–45 with PMS, and the yoga group performed it for 10 weeks in 3 sessions with each session of 60 min duration. As a result, they highlighted that yoga significantly relieves the PMS symptoms [12]. These two studies have been focused on exercises of different kinds and intensities.

Recently, physical activity (PA) and its ability to prevent menstruation-related symptoms have attracted the attention of experts and women [13]. PA is an activity that involves bodily movements resulting in energy expenditure, whereas exercise is a subcategory of PA [14]. Only a few reports focused on the relationship between PMS and the amount of PA, including not only exercise but also routine movement. One study examined PA’s self-report measures using questionnaires, diaries, and brief logs [15]. However, only a few reports measured the regular PA in the daily lives of women in their childbearing age [16]. This vital information can provide a more detailed interpretation of the results.

International public health guidelines for adults recommend 150 min of moderate-intensity PA per week, equivalent to 600 MET-minutes/week (MET-minutes is a unit that expresses PA quantity) [17, 18]. A scoping review of physical activity among college students reported that 58.7% of students met physical activity recommendations equivalent of ≥ 600 MET-minutes/week, but physical activity levels in college students were said that remain concerningly low [19]. Moreover, the Japanese guidelines on health promotion and recommendations for PA and exercise shows that every adult should accumulate 1380 MET-minute/week of PA at an intensity level of at least 3 METs to prevent chronic diseases and to obtain numerous health benefits [20]. These are the recommended PA values for 18–64 years. A higher PA setting, at least 1380 MET-minutes/week or more, might be necessary for young women who are highly active and have high metabolisms to ensure their continued health. In previous studies, higher levels of sports and physical activity have also been suggested to be associated with more positive mental health and quality of life in college students [21, 22]. Thus, it may be meaningful for female college students to focus on high PA.

In addition, most of the studies [23] encouraged the employment of regular, moderate-intensity aerobic exercise [24] as a potential intervention for preventing PMS. Moderate-intensity aerobic exercises corresponds to high PA in daily life. Thus, we focused on high PA to set more age-appropriate quantities of PA for women of childbearing age. The purpose of this study was to investigate the relationship between PMS symptoms and high PA in female college students. We hypothesised that PMS symptoms were milder in those with a high PA.

## Methods

This cross-sectional study designed to determine the relationship between PA and PMS symptoms was conducted following the Helsinki Declaration of 1975, as revised in 1983. This study was also approved by the Kyoto University Graduate School and Faculty of Medicine and Kyoto University Hospital Ethics Committee (approval number: R1442). The participants received information regarding our research and provided written or verbal consent after understanding the study protocol. All information regarding participant data remains confidential. The participants’ responses were anonymous.

### Participants

In this study, 427 women consented to participate. This study was conducted in Japan between August and September 2016. The participants included in this study were a convenience sample consisting of undergraduate and graduate female students from several Japanese universities. Participants in this study included both those who belonged to sports clubs (more than 20 different sports) and those who did not. The inclusion criteria were as follows: affiliated with a university in Japan, literate in Japanese so as to read and understand the proposed survey, potentially reproductive, and consented to participate. Those who were excluded from the study were menopausal women, those with current depression, anxiety, or any other psychiatric disorder previously diagnosed, those who received hormonal therapy, those who experienced a traumatic life event (widowhood, death of a close friend or relative, and imprisonment) before or during the study period, and those who were not due to drug therapy, hormonal internal use, or drugs.

### Questionnaire

Our survey was administered via a paper or a web-based questionnaire with the same content. The questionnaire included basic information such as age, height, weight, menstrual conditions, levels of PMS symptoms, and PA.

#### PMS

PMS symptoms were referred to as premenstrual dysphoric disorder (PMDD) (An additional movie file shows this in more detail [see Additional file 1]) in accordance with the American Psychiatric Association, 5th edition of the Diagnostic and Statistical Manual of Mental Disorders [25] and as PMS symptoms in the American College of Obstetrics and Gynecology guidelines (Additional file 2). We identified the symptoms among our participants by adding them in the questionnaire. We based our questionnaire on a previous study [26]. The symptoms we added were ‘Lower abdominal pain’, ‘Increased appetite’, ‘Easily fatigued and feeling listless’, ‘Low back pain’, ‘Sleepiness’, ‘Decreasing concentration’, and ‘Fatigue or lack of energy Overeating’. The total number of PMS items in the questionnaire was 18 (Additional file 3).

To establish a diagnosis of PMS based on previous guidelines [27], we included a question regarding the presence of the symptoms in the past three menstrual cycles and whether at least one of the physical or psychological symptoms were observed 5 days prior to menstruation. The participants answered a questionnaire based on self-review. In this study, among all the items regarding symptoms, participants who checked as ‘No problem but symptomatic’ or ‘Problematic’ for one or more items were diagnosed with PMS.

All symptoms were separated into three levels. The ‘problematic symptoms’ corresponded to symptoms interfering with activities of daily living. ‘No problem but symptomatic’ corresponded to symptoms not interfering with activities of daily living. The final category, the ‘no symptom’, corresponded to the lack of symptoms.

The total score was calculated according to the following: no symptoms, 0 points; no problem but symptomatic, 1 point; and problematic symptoms, 2 points (Additional file 2).

For each symptom, we divided the patients into two groups: participants with and without symptoms.

#### PA

The International Physical Activity Questionnaire (IPAQ) is the most representative questionnaire for assessing PA. Several studies have reported using the IPAQ to measure PA [28, 29]. The IPAQ can be applied to participants of a wide age range. Its reliability is reportedly very good, while its validity is comparable to those of most other self-report questionnaires [30]. Moreover, they have reasonable measurement properties for monitoring the levels of PA among a population consisting of 18–65-year-old adults with diverse backgrounds [30].

We used the short version of the IPAQ to determine the participants’ weekly PA. We asked about participants’ moderate intensity active time, vigorous-intensity active time, and walking time. The participants’ METs were then calculated according to the IPAQ guidelines [31]. These values were calculated as follows:

$$Walking \,MET-\frac{minutes}{week}= 3.3 * walking \,minutes * walking \,days$$

$$Moderate\, MET-\frac{minutes}{week}= 4.0 * moderate-intensity \,activity \,minutes * moderate-intensity \,days$$

$$Vigorous \,MET-\frac{min}{week}= 8.0 * vigorous-intensity \,activity \,minutes * vigorous-intensity \,days$$

$$Total \,physical \,activity \,MET-minutes/week = sum \,of \,walking + moderate + vigorous \,MET- minutes/week scores$$

In the IPAQ guidelines, a separate category labelled ‘high’ can be computed to describe higher levels of PA participation. The criteria for classification as ‘high’ are seven or more days of any combination of walking, moderate-intensity, or vigorous-intensity activities achieving a minimum total PA of at least ≥ 3000 MET-minutes/week [31]. The recommended PA value in Japan is 1380 MET-minutes/week, but NIH guidelines report that doubling the time may increase health benefits [32]; therefore ≥ 3000 MET minutes/week, which represents high physical activity in the IPAQ guidelines, may be an appropriate index.

### Statistical analyses

The PA data were divided into two groups according to the total metabolic equivalent of task (MET) in a week, which was defined by IPAQ as ≥ 3000 MET-minutes/week and < 3000 MET-minutes/week). The two groups’ severity scores for each of the PMS physical and psychological symptoms calculated based on the questionnaire were compared. Associations with characteristics were assessed using the Wilcoxon rank-sum test. After observing a significant difference in this analysis, we performed a multivariate logistic regression analysis using Body Mass Index (BMI) as adjustment variable [28]. We then divided the participants based on the presence or absence of each symptom using the chi-square test for comparison in terms of the IPAQ. The statistical significance level was set at p < 0.05. All analyses were conducted using the JMP Pro 14.0 (SAS Institute, Cary, NC, USA).

## Results

The questionnaire was completed by a total of 381 female university students (89.23%). The 46 women (10.77%) for whom questionnaires were incomplete were excluded from the analysis. The participants’ characteristics are described in Tables 1 and 2 (mean ± SD, age: 20.4 ± 1.2 years; height: 159.5 ± 5.6 cm; weight: 51.7 ± 5.8 kg; BMI: 20.27 ± 1.78). Of these, 76.3% had regular menstrual cycles (25–38 days), while 90.2% students had a PMS.

**Table 1:** Basic information of participants

	Total (n = 381)
Age (y)	20.44 ± 1.23
Height (cm)	159.57 ± 5.58
Weight (kg)	51.67 ± 5.75
BMI (kg/m^2^)	20.27 ± 1.78
Number of the women belonging to sports club [n (%)]	211 (55.38)
Total PMS score	8.84 ± 6.89
Energy expenditure (kcal/week)	4448.90 ± 4305.07
Total physical activity (MET-minutes/week)	4829.09 ± 4537.48

**Table 2:** Menstrual information of participants

	n = 381(%)
*Regular menstrual cycles*	
Yes	291 (76.38)
No	90 (23.62)
*PMS*	
Yes	344 (90.29)
No	37 (9.71)

The results are shown in Table 3, the participants were divided into two groups based on their total PA. Those with total PA of ≥ 3000 MET-minutes/week had lower total PMS symptom scores (*p* < 0.01), physical symptom scores (*p* = 0.01), and psychological symptom scores (p = 0.01).

**Table 3:** The Wilcoxon's rank-sum test of group divided based on those who met at least ≥ 3000 MET-minutes/ week and score of PMS symptoms

	Total physical activity (MET-minutes/week)			
	≥ 3000 (n = 200)	< 3000 (n = 181)	z	p-value
Physical symptoms	4.94	5.79	2.66	0.01
Psychological symptoms	3.21	3.99	2.47	0.01
PMS symptoms	8.16	9.78	2.88	< 0.01

Table 4 shows the result of the multivariate logistic regression analysis with BMI as adjustment variable. There were significant associations between PMS symptoms (physical and psychological) and a total PA of ≥ 3000 MET-minutes/week.

**Table 4:** Multivariate logistic regression analysis with BMI as an adjustment variable

	Total physical activity (≥ 3000MET-minutes/week)		
	Odds ratio	95%CI	p-value
Physical symptoms	0.94	(0.88–0.99)^a^	0.02
Psychological symptoms	0.95	(0.89–1.00)^a^	0.05
PMS symptoms	0.97	(0.93–0.99)^a^	0.02

*CI* Confidence Intervals

^a.^In the 95% CI, values beyond third decimal places are rounded down

For each PMS symptom, we observed differences in the proportion of the presence or absence of some PMS symptoms between the two groups classified as ≥ 3000 MET-minutes/week and < 3000 MET-minutes/week. As Table 5, a high proportion of participants who with ≥ 3000 MET -minutes/ week had none of these symptoms is high: ‘sleepiness’, ‘acne’, ‘lower abdominal pain, ‘feeling depressed’, ‘decreasing concentration’, ‘easily fatigued and feeling listless’.

**Table 5:** The proportion of those with symptoms in the group divided based on the total METs

	Total physical activity (MET-minutes/week)		
With symptoms	≥ 3000 (n = 200)	< 3000 (n = 181)	p-value
Sleepiness	103 (27.03)	124 (32.55)	< 0.01
Acne	61 (16.01)	80 (21.00)	< 0.01
Lower abdominal pain	106 (53.00)	117 (64.64)	0.02
Feeling depressed	84 (22.05)	95 (24.93)	0.04
Decreasing concentration	66 (17.32)	87 (22.83)	< 0.01
Easily fatigued and feeling listless	84 (22.05)	96 (25.20)	0.03

n (row%). No significant associations were found for other symptoms. Chi-square test

## Discussion

In this study, we found that highly PA women had lower PMS symptom scores (physical and psychological symptoms). This study clarified two results. First, people with a PA of ≥ 3000 MET-minutes/week had lower scores for psychological and physical symptoms. Second, a higher proportion of participants with no symptoms were found among those met ≥ 3000 METs/week in several PMS symptoms.

PMS is one of the most common problems among women. Moreover, PMS can interfere with activities of daily living. Thus, finding a way to prevent or treat PMS is an important health and research priority. In our study, we found that over 90% of women reported at least one symptom of PMS. This finding was similar to a study that found that 95% of participating women aged 18–24 years experienced at least one PMS symptom [33].

Our first conclusion was that people whose PA is ≥ 3000 MET -minutes/week had lower scores in psychological and physical symptoms. This conclusion complemented Koushkies et al.’s finding, which showed an association between an increased PA and a significant reduction in PMS symptoms [34]. However, Kroll-Desrosiers et al.’s study showed no evidence of an association between PA and either the presence of PMS or premenstrual symptom severity [16]. The difference must have been due to the difference in method. Kroll-Desrosiers et al.’s examined PA by continuous METs per week and analysed the data by dividing the group by tertiles of PA. Higher levels of participation were characterised using the IPAQ, which defined vigorous-intensity activity ≥ 3000 MET-minutes/week. Though the difference in PMS symptom scores is about 1 point in our result, a 1-point improvement in our research assessment indicates either "one less PMS symptom" or "a certain PMS symptom is no longer enough to interfere with life" and this improvement is influenced to PMS severity and diagnosis. A previous study has been reported that physical functioning, physical role, general health, social functioning, mental health, and vitality scores significantly decreased as the severity of PMS symptoms increased in university students [35]. Thus, 1-point differences in this study for PMS score could be clinically important in young women.

Regarding the second conclusion, the ratio of participants who had ≥ 3000 MET-minutes/week had no symptoms. The symptoms we found in this study were ‘sleepiness’, ‘acne’, ‘lower abdominal pain, ‘feeling depressed’, ‘decreasing concentration’, and ‘easily fatigued and feeling listless’. Some studies explained this phenomenon. Wilmore et al. showed that regular PA had many benefits, including reduction of stress and PMS [36]. Additionally, PA might still affect sex hormone function by modulating target tissue sensitivity to these hormones [37]. A previous study showed a significant decrease in prolactin, oestradiol, and progesterone levels, resulting in fatigue, impaired concentration, confusion, and most PMS symptoms. In addition, a study showed that increased prolactin level in the late luteal phase was one of the causes of breast pain and swelling. PA in non-athletes possibly reduces the status of this hormone; thus, the ratio of breast pain and swelling may be reduced. However, our results did not corroborate with this finding [38]. PA, including resistive exercise, induces neuroimmunomodulation effects, increases neurotrophies [39]and β-endorphins [40], decreases the sympathetic response, affects the hypothalamic–pituitary–adrenal axis reactions, and improves the serotonin system. All of these responses may decrease anxiety and depression [41].

Acne is a symptom associated with polycystic ovarian syndrome and is a common metabolic disturbance, particularly in women [42, 43]. A study suggested that regular exercise decreased plasma insulin levels and reduced metabolic disease [44]. Sleep and activity during awake time interact to influence many aspects of health [45]. Moreover, a study showed that age and sex were associated with PA and sleep. They found that aspects of PA were significantly lower in younger adults (20–39 years old) who reported more frequent daytime sleepiness [46].

Priya et al. studied the effect of aerobic exercise at different intensities on PMS and concluded that moderate-intensity aerobic exercise should be encouraged as a potential prophylaxis for PMS [47]. This study gave us a range of PAs that they investigated. In this study, we converted different intensities of exercise into numbers that were calculated from the participants’ weekly PA. We were not simply considering exercise alone, but also the overall PA. In addition to high-intensity exercise in participants engaged in sports, we also examined the presence of routine moderate-intensity exercise and walking time. More than half (55.4%) of the participants in this study belonged to sport clubs, and 52.5% met high PA. A previous study reported that 21.2% of Japanese aged 18–39 years met high PA [48]. While the percentage in this study is not necessarily high because 47% reported a decrease in PA after graduating from college compared to during college [49], it is possible that the population was as a whole more active than the general younger population. Moreover, this study focused on meeting high total PA regardless of the type of PA intensity. Future research may be needed to examine the relationship between the type of PA intensity and PMS symptoms, for example, which type of PA intensity constitute a weekly high PA, whether there is a type of PA intensity that is most effective in reducing PMS symptoms, and whether there is an appropriate duration of activity for each type of PA intensity, which would provide more useful information.

In addition, we considered that PMS symptoms affected routine PA. A systematic review and meta-analysis indicated that exercise performance might be trivially reduced during the early follicular phase of the menstrual cycle compared with all other stages [23]. We considered the possibility that PMS symptoms affected exercise. Although some of the participants’ PA included high-intensity exercise in our study, it could not be denied that PMS affected PA. Although this study focused on high PA, inactivity is also a problem and may affect variety health problems, including PMS symptoms [50]. Thus, PA may be impaired in those with high PMS severity scores, making it necessary to focus on physical inactivity along with high PA.

In summary, according to our results, female students with ≥ 3000 MET-minutes/week of PA had milder symptoms of PMS.

### Study limitations

Researchers frequently rely on questionnaires to assess PA because of their low cost and ease of administration. However, inherent limitations, such as participant recall bias and an inability to accurately retrospectively recall relevant PA details, may lead to overestimation or underestimation of PA. The target is limited, and causality is not known. The IPAQ-short version typically overestimates PA as measured by an objective criterion by an average of 84% [51]. In addition, the research population in this study may have been selected through the collection of web and paper-based questions. Therefore, they may have met higher PA levels than the general younger population. Additionally, the target population was limited to Japanese university students. Therefore, it is possible that the population was younger in age and relatively high in socioeconomic status [52]. Therefore, it should be noted that the results may not be adaptable to other populations. Furthermore, the data were collected in August and September, a time of relatively high activity [53]. In Japan, which has four seasons, PA is highly influenced by the season, so the limited season of this study is also considered a limitation of this study. Finally, this study was selected 3000 MET min/week as the index of high physical activity using the IPAQ guidelines. However, the rationale for 3000 MET min/week as a particular cut-off is still unclear. Further detailed studies are needed in the future to indicate a more appropriate cut-off value for PA for young women.

## Conclusion

This study is one of the few studies that analysed the relative factors of PMS based on regular PA using a questionnaire in female university students. The findings of this research showed that young women with high PA of ≥ 3000 MET-minutes/week had milder physical and psychological symptoms of PMS than women with PA of < 3000 MET-minute/week. This may indicate that high PA may be an important factor in PMS severity in young women as college students. Further fture intervention studies are needed to provide a potential new non-pharmacological management of PMS using PA as high as ≥ 3000 MET-minutes/week as an index of PA in female students. Active promotion of PA in daily life may have a positively influence on the health problems including PMS symptoms of young women.

## Supplementary Information

**Additional file 1**. Premenstrual syndrome (PMS) diagnostic criteria of the American College of Obstetrics and Gynecology (2000).

**Additional file 2**. PMS symptoms questionnaire.

**Additional file 3**. Questionnaire of diagnostic criteria for premenstrual dysphoric disorder.

## Data Availability

Data cannot be shared publicly because of the policy of the Kyoto University for data safety and protection. Data are available on request from the faculty of human health sciences for researchers who meet the criteria for access to confidential data or enrolled in the faculty. Data requests may be directed to the Kyoto University Graduate School and Faculty of Medicine, Ethics Committee, Department of Ethics Support (email: ethcom@kuhp.kyoto-u.ac.jp; telephone/fax: 0081-75-753-4680). Data requests may also be directed to Assistant Professor Momoko Tanima, who is in the department and unaffiliated with the current study (email: tanima.momoko.8 s@kyoto-u.ac.jp; telephone: 0081-75-751-3964).

## Abbreviations

PMS: Premenstrual syndrome
PMDD: Premenstrual dysphoric disorder
bpm: Beats per minute
PA: Physical activity
IPAQ: International Physical Activity Questionnaire
MET: The Metabolic Equivalent of Task
BMI: Body Mass Index
