# Supplementary material for: The relationship between high physical activity and premenstrual syndrome in Japanese female college students
Source: BMC Sports Sci Med Rehabil. 2022 Sep 26;14:175. doi: 10.1186/s13102-022-00569-0 (PMC9511710; doi:10.1186/s13102-022-00569-0)
Supplement: Supplementary file 1 — Additional file 1. Premenstrual syndrome (PMS) diagnostic criteria of the American College of Obstetrics and Gynecology (2000). [file 13102_2022_569_MOESM1_ESM.docx]

Additional file 1. Premenstrual syndrome (PMS) diagnostic criteria of the American College of Obstetrics and Gynecology (2000).

| In the past three menstrual cycles, at least one of the following physical symptoms or psychological symptoms were observed during the 5 days before menstruation. | | | |
| --- | --- | --- | --- |
|  | Psychological symptoms | Physical symptoms |  |
|  | Depression | Breast tenderness |  |
|  | Anger | Abdominal distension |  |
|  | Irritability | Headache |  |
|  | Anxiety | Limbs swelling |  |
|  | Confusion |  |  |
|  | Absent from society |  |  |
| These symptoms were relieved within 4 days after the start of menstruation. They did not recur in the next 13 days after the onset of menstruation. | | | |
| These symptoms were not due to drug therapy, hormonal internal use, drugs, or alcohol use. | | | |
| The symptoms must occur reproducibly during the two cycles of prospective recording. | | | |
| A clear obstacle of social or economic competence was recognised. | | | |
