# Supplementary material for: The relationship between high physical activity and premenstrual syndrome in Japanese female college students
Source: BMC Sports Sci Med Rehabil. 2022 Sep 26;14:175. doi: 10.1186/s13102-022-00569-0 (PMC9511710; doi:10.1186/s13102-022-00569-0)
Supplement: Supplementary file 2 — Additional file 2. PMS symptoms questionnaire. [file 13102_2022_569_MOESM2_ESM.docx]

Additional file 2. PMS symptoms questionnaire

|  | Symptoms | No symptoms | No problem but symptomatic | Problematic |
| --- | --- | --- | --- | --- |
| Physical symptoms | Breast tension and pain | □ | □ | □ |
|  | Headache | □ | □ | □ |
|  | Lower abdominal tightness | □ | □ | □ |
|  | Lower abdominal pain | □ | □ | □ |
|  | Swelling of hands and feet | □ | □ | □ |
|  | Increased appetite | □ | □ | □ |
|  | Easily fatigued and feeling listless | □ | □ | □ |
|  | Low back pain | □ | □ | □ |
|  | Sleepiness | □ | □ | □ |
|  | Acne | □ | □ | □ |
| Mental symptoms | Feeling depressed | □ | □ | □ |
|  | Feeling irritable | □ | □ | □ |
|  | Feeling confused | □ | □ | □ |
|  | Having feelings of sudden anger | □ | □ | □ |
|  | Feeling anxious | □ | □ | □ |
|  | A desire to be alone | □ | □ | □ |
|  | Fatigue or lack of energy Overeating | □ | □ | □ |
|  | Decreasing concentration | □ | □ | □ |
| □symptoms not applicable to the above. （for example： ) | | | | |
