# Supplementary material for: The relationship between high physical activity and premenstrual syndrome in Japanese female college students
Source: BMC Sports Sci Med Rehabil. 2022 Sep 26;14:175. doi: 10.1186/s13102-022-00569-0 (PMC9511710; doi:10.1186/s13102-022-00569-0)
Supplement: Supplementary file 3 — Additional file 3. Questionnaire of diagnostic criteria for premenstrual dysphoric disorder. [file 13102_2022_569_MOESM3_ESM.docx]

Additional file 3　Diagnostic Criteria for Premenstrual Dysphoric Disorder

| A. In the majority of menstrual cycles, at least five symptoms must be present in the final week before the onset of menses. These symptoms should have started to improve within a few days after the onset of menses and should have improved or absent in the week after menses. | |
| --- | --- |
| B. One (or more) of the following symptoms must be present: | |
|  | 1. Marked affective lability (e.g., mood swings; feeling suddenly sad or tearful, or increased sensitivity to rejection). |
|  | 2. Marked irritability or anger or increased interpersonal conflicts. |
|  | 3. Marked depressed mood, feelings of hopelessness, or self-deprecating thoughts. |
|  | 4. Marked anxiety, tension, and/or feelings of being keyed up or on edge. |
| C. One (or more) of the following symptoms must additionally be present, to reach a total of five symptoms when combined with symptoms from Criteria B, which can be seen above. | |
|  | 1. Decreased interest in usual activities (e.g., work, school, friends, hobbies). |
|  | 2. Subjective difficulty in concentration. |
|  | 3. Lethargy, easy fatigability, or marked lack of energy. |
|  | 4. Marked change in appetite; overeating; or specific food cravings. |
|  | 5. Hypersomnia or insomnia. |
|  | 6. A sense of being overwhelmed or out of control. |
|  | 7. Physical symptoms such as breast tenderness or swelling, joint or muscle pain, a sensation of “bloating,” or weight gain. |
| note: *The symptoms in Criteria A–C must have been met for most of the menstrual cycles that occurred in the preceding year*. | |
| D. The symptoms are associated with clinically significant distress or interference with work, school, usual social activities, or relationships with others (e.g., avoidance of social activities; decreased productivity and efficiency at work, school, or home). | |
| E. The disturbance is not merely an exacerbation of the symptoms of another disorder, such as major depressive disorder, panic disorder, persistent depressive disorder (dysthymia), or a personality disorder (although it may co-occur with any of these disorders). | |
| F. Criterion A should be confirmed by prospective daily ratings during at least two symptomatic cycles. (note: The diagnosis may be made provisionally before this confirmation.) | |
| G. The symptoms are not attributable to the physiologic effects of a substance (e.g., a drug of abuse, a medication, other treatment) or another medical condition (e.g., hyperthyroidism). | |

*Reprinted with permission from the American Psychiatric Association*. Diagnostic and Statistical Manual of Mental Disorders. *5th ed. Washington DC: American Psychiatric Association; 2013:171–172*.
